# The Medical Profession and the Public Press

**Published:** 1852-03

**Authors:** 


### ARTICLE IV.

### THE MEDICAL PROFFSSION AND PUBLIC PRESS.

It is a lamentable fact that the great lever by which public opinion in this country is formed and controlled, the public press, has to a great extent, been suffered to pass into the interests of quackery.

Articles, both editorial and communicated, noticing the different special systems of medical practice, and nostrums, and men connected with them,as well as crowded columns of their advertisements are continually making their appearance in the columns of the news-papers, while the notices of scientific improvements, the transactions of the regular profession and of the men who so devotedly labor in its ranks, are very rarely to be seen. And when these are noticed, it is too often in a manner to do the profession more injury than good.

That this prostitution of the public press to the service of the quack need not to be so, is written in the fact that the regular profession are more numerous and more influential, except in their having almost wholly lost this arm of power.

Shall we not see to it, that our conventions, society meetings, discoveries and public movements, with biographical sketches of our distinguished men receive more attention in the public journals? We would be far from approving of individal puffs, which are undignified and ought not to be sought for, or countenanced by honorable physicians; but certainly a knowledge of the resources of our art, and the utility of our practice, must by some means be kept before the pnblic, or our science will be of no avail, and our skill will neither be appreciated nor called into requisition. And shall we disregard the public press, the most influential of the means for accomplishing this end ? Shall we wrap ourselves up in our dignity, and by a stollid indifference to the education of the public in such matters, say we are wise and skilful, if you only knew it, but will not deign to tell of our wisdom, nor to bring to your knowledge the evidence of our skill ? Certainly all experience, all success, admonishes us to pursue a different policy.

Let us then, exert our influence to secure a full amount of attention to the profession by the public journals, by writing for, and aiding and patronizing such as publish scientific articles and liberal notices of the regular profession, and great good to the community will be the result.

